# Genome-wide identification of binding sites for NAC and YABBY transcription factors and co-regulated genes during soybean seedling development by ChIP-Seq and RNA-Seq

**DOI:** 10.1186/1471-2164-14-477

**Published:** 2013-07-16

**Authors:** Md Shamimuzzaman, Lila Vodkin

**Affiliations:** 1 Department of Crop Sciences, University of Illinois, Urbana, IL 61801, USA

## Abstract

**Background:**

Two plant-specific transcription factors, NAC and YABBY, are involved in important plant developmental processes. However their molecular mechanisms, especially DNA binding sites and co-regulated genes, are largely unknown during soybean seedling development.

**Results:**

In order to identify genome-wide binding sites of specific members of the NAC and YABBY transcription factors and co-regulated genes, we performed Chromatin Immunoprecipitation Sequencing (ChIP-Seq) and RNA Sequencing (RNA-Seq) using cotyledons from soybean seedling developmental stages. Our RNA-Seq data revealed that these particular NAC and YABBY transcription factors showed a clear pattern in their expression during soybean seedling development. The highest level of their expression was found in seedling developmental stage 4 when cotyledons undergo a physiological transition from non-photosynthetic storage tissue to a metabolically active photosynthetic tissue. Our ChIP-Seq data identified 72 genes potentially regulated by the NAC and 96 genes by the YABBY transcription factors examined. Our RNA-Seq data revealed highly differentially expressed candidate genes regulated by the NAC transcription factor include lipoxygense, pectin methyl esterase inhibitor, DEAD/DEAH box helicase and homeobox associated proteins. YABBY-regulated genes include AP2 transcription factor, fatty acid desaturase and WRKY transcription factor. Additionally, we have identified DNA binding motifs for the NAC and YABBY transcription factors.

**Conclusions:**

Genome-wide determination of binding sites for NAC and YABBY transcription factors and identification of candidate genes regulated by these transcription factors will advance the understanding of complex gene regulatory networks during soybean seedling development. Our data imply that there is transcriptional reprogramming during the functional transition of cotyledons from non-photosynthetic storage tissue to metabolically active photosynthetic tissue.

## Background

Understanding the transcriptional regulatory network during developmental stages has been a focus for many years at the single gene level. Recently genome-wide mapping of protein–DNA interactions enables of complete understanding of transcriptional regulation. A precise map of binding sites for transcription factors and other DNA-binding proteins is vital for deciphering the gene regulatory networks that underlie various biological processes [1-4]. Transcription factors bind to DNA sequences and regulate gene expression both in animals and plants [5-8]. Virtually all biological processes are directly regulated or influenced by transcription factors. More specifically, developmental processes are highly regulated by the appearance and disappearance of particular transcription factors [5,7-9]. Therefore, the identification of transcription factor binding sites has immense importance for unraveling the gene regulation during developmental stages.

To study the genome-wide profiling of transcription factor binding sites, chromatin immunoprecipitation followed by sequencing (ChIP–Seq) has become one of the principal techniques [1,2,10]. Owing to the tremendous progress in next-generation sequencing technology, ChIP–Seq offers higher resolution, less noise and greater coverage than its array-based predecessor ChIP–chip [11,12]. With the decreasing cost of sequencing, ChIP–Seq has become an indispensable tool for studying gene regulation. It has been widely used to study transcription factor binding, histone modifications and DNA methylation [4,13]. The combination of ChIP-Seq and quantitative measurements of transcriptomes (RNA-Seq) is increasingly used to decipher the regulation of gene expression by transcription factors [10,14].

**Figure 1:**
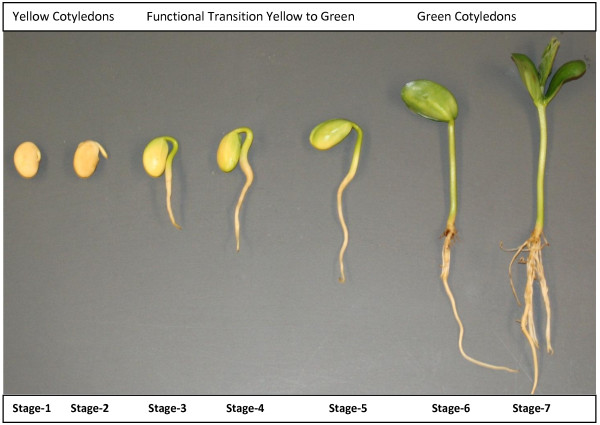
**Seven different developmental stages of soybean seedling development.** Seven different stages during the development of soybean seedlings are defined based on time, size of radicles, hypocotyls, roots and appearance of germinating cotyledons. Stage 1: Imbibed seed for 24 hours; pre-emerging hypocotyls. Stage 2: Yellow cotyledons; emerging radicle 8–10 mm long. Stage 3: Yellow cotyledons with slightly green edges; 15–20 mm long hypocotyls. Stage 4: Yellow-green cotyledons; hypocotyls 30–35 mm long. Stage 5: Yellow-green cotyledons above the ground; primary roots starting to develop. Stage 6: Mostly green cotyledons above the ground; growing straight from the hypocotyl. Stage 7: Fully green cotyledons; plants 6–7 cm long above the ground; the root system fully developed; cotyledons upright; unifoliolate exposed.

Soybean (*Glycine max*) is one of the most economically important crops cultivated all over the world. It is an excellent source of vegetable oil and protein. However, the regulation of gene expression during soybean seedling development is still largely unexplored. Thus it is important to investigate how the genes are regulated during different developmental stages of soybean seedlings. The soybean genome was sequenced about two years ago [15] and this information has widened the molecular research on soybean. Soybean gene annotation revealed a number of transcription factors and many of them play vital roles during seedling development [8,16]. Seven different stages of soybean seedling development were based on time, size of radicles, hypocotyls, roots, emergence and development of unifoliolate, and appearance of germinating cotyledons (Figure 1). It has been reported that changes in developmental stages shift the level of gene expression in cotyledon of germinating soybean seedlings [17] and other developmental factors [8]. These changes are accomplished by the changes in the expression level of transcription factors [5,8,9]. A number of soybean developmental stage associated transcription factors have already been reported, including NAC , SNF2, zinc finger transcription factor , transcription factor homolog BTF3-like protein and transcription repressor ROM1 [17,18]. To deeply investigate the transcription factor mediated regulation of gene expression during soybean seedling development, we constructed ChIP-Seq and RNA-Seq libraries using developmental stage specific cotyledons. There is a functional transition between developmental stage 4 and stage 5 when cotyledons undergo a physiological transition from mainly a nutrient and food reserve tissue (yellow) to an active photosynthetic (green) tissue. The physiological transition of the cotyledon is a complex process that must be under strict gene control and regulation. To investigate the expression level of transcription factors and co-regulated genes, we constructed seven different RNA-Seq libraries specific to each developmental stage. Based on our RNA-Seq data, we selected specific NAC and YABBY transcription factors which showed a clear pattern in their expression from lower level to higher level during the physiological transition of soybean seedlings.

These two transcription factors, NAC and YABBY, are involved in numerous biological processes [16,19,20]. The NAC family (NAM/ATAF/CUC) constitutes a highly prolific group of plant-specific TFs, with representatives in monocots, dicots, conifers and mosses [21,22]. Many plants of commercial and scientific interest, such as *Glycine max*, have more than 100 different NAC proteins [16,23]. In this study, we focused on four specific genes encoding NAC transcription factors which showed a clear expression pattern during seedling development. NAC transcription factors play important roles in plant growth, development, and stress responses [23-25]. Previously it was shown in *Arabidopsis* that a consensus DNA sequence (CGT[GA]) to which NAC and other relatively distant NAC TFs bind [26]. But there is no experimental evidence in legumes for their DNA binding sites and co-regulated genes.

Another group of plant specific transcription factors is YABBY, which plays a critical role in determining organ polarity [27-30]. It is involved in the establishment of abaxial-adaxial polarity in lateral organs [19,20,31]. YABBY family transcription factors contain a zinc-finger domain in the amino-terminal region and a YABBY domain in the carboxyl-terminal region [19,20,32]. There is not much known about the molecular mechanisms of this YABBY transcription factor including the DNA binding sites and co-regulated genes during legumes development.

In order to dissect the developmental stage specific gene regulation by NAC and YABBY transcription factors, we developed polyclonal antibodies against synthetic peptides representing specific members of those transcription factors and performed ChIP-Seq experiment using pooled cotyledons from stage 4 and stage 5. We investigated the differential expression of gene models identified by ChIP-Seq between stage 3 (before the functional transition) and stage 6 (after the functional transition) using our RNA-Seq data. Our ChIP-Seq data identified many candidate genes that are regulated by specific members of NAC and YABBY transcription factors. Motif analysis discovered three separate motifs for NAC and YABBY transcription factors. RNA-Seq analysis revealed the expression in reads per kilobase of gene model per million mapped reads (RPKM) for these motif-containing Glyma models during different developmental stages. The expression analysis efficiently identified differentially expressed genes between stage 3 and stage 6. The identification of NAC and YABBY transcription factor binding sites and the potential genes regulated by these transcription factors will advance our understanding of gene regulation during legume development.

## Results

### RNA-Seq reveals the differential expression of NAC and YABBY transcription factor

**Figure 2:**
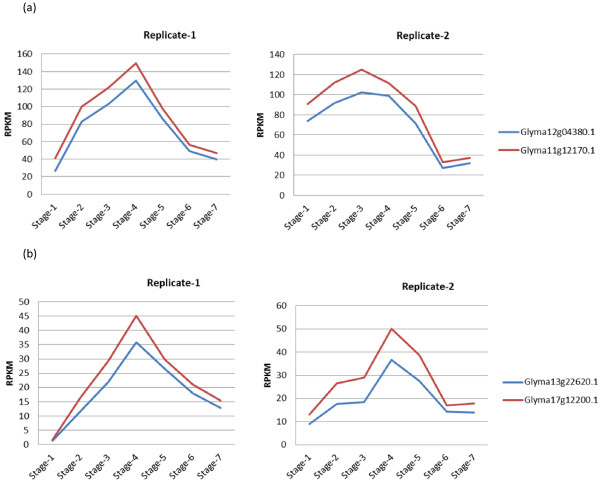
**Expression level of NAC and YABBY transcription factor during seven developmental stages of soybean seedling.** The graph shows a clear expression pattern for NAC and YABBY transcription factors during soybean seedling developmental stages. **(a)** NAC family transcription factor expression level measured by RNA-Seq during seedling development. **(b)** YABBY transcription factor expression level measured by RNA-Seq during seedling development. Their expression gradually increased from germinating cotyledons stage 1 to stage 4. The highest level of expression is found at stage 4. Then it gradually decreased as the germinating cotyledons are advanced to the subsequent stages. RNA-Seq data has been normalized to reads per kilobase of gene model per million mapped reads (RPKM) values and we have made two replicates of each stage.

To understand the molecular mechanisms involved in the functional transition during soybean seedling development, we constructed seven different RNA-Seq libraries using cotyledons from each developmental stage separately. High throughput sequencing-by-synthesis (SBS) of these libraries produced 46 million to 76 million reads. Most of these reads mapped to the soybean reference genome and transcriptome of cv. Williams 82 available at the Phytozome database [33]. Analysis of RNA-Seq data from different developmental stages revealed the differential expression of many genes including transcription factors. In this study, we focused on NAC and YABBY transcription factors which showed a clear expression pattern during soybean seedling development (Table 1 and Figure 2). Their expression gradually increases from stage 1 to stage 4 of soybean germinating cotyledons. The highest level of expression was found at stage 4. Then it gradually decreased as the germinating cotyledons develop a mature seedling. There are four specific members of NAC family and two specific members of YABBY family showed this expression pattern. The expression pattern is shown graphically for two specific members of NAC family among four and two specific members of YABBY family (Figure 2). There was a 5 to 10-fold range in differences of RPKM values between stage 1 to stage 4 and stage 5 (Table 1 and Figure 2).

**Table 1:** RNA-Seq reveals the differential expression of two transcription factors (TF) in germinating cotyledons

				**RPKM**				
**Gene model (R)**	**S-1**	**S-2**	**S-3**	**S-4**	**S-5**	**S-6**	**S-7**	**Annotation**
Glyma12g04380.1 (R1)	26.8	82.5	103	129	85.9	49.0	39.6	NAC Transcription Factor
Glyma12g04380.1 (R2)	73.8	91.8	102	99.1	71.3	27.2	31.8	NAC Transcription Factor
Glyma11g12170.1 (R1)	41.1	99.7	121	149	97.6	56.2	46.7	NAC Transcription Factor
Glyma11g12170.1 (R2)	90.9	111	124	111	88.7	32.7	37.2	NAC Transcription Factor
Glyma11g12180.1 (R1)	35.9	95.4	117	146	96.2	55.1	45.9	NAC Transcription Factor
Glyma11g12180.1 (R2)	87.1	108	122	108	86.0	33.2	37.8	NAC Transcription Factor
Glyma01g18780.1 (R1)	8.56	31.7	43.5	68.0	49.5	34.3	18.9	NAC Transcription Factor
Glyma01g18780.1 (R2)	22.1	39.3	41.5	57.3	41.4	20.3	21.4	NAC Transcription Factor
Glyma13g22620.1 (R1)	1.2	11.6	21.9	35.7	26.5	17.9	12.7	YABBY Transcription Factor
Glyma13g22620.1 (R2)	8.8	17.6	18.4	36.5	27.5	14.3	13.8	YABBY Transcription Factor
Glyma17g12200.1 (R1)	1.67	16.3	29.3	45.2	29.8	21.0	15.4	YABBY Transcription Factor
Glyma17g12200.1 (R2)	13.0	26.4	28.9	50.1	38.7	16.9	17.7	YABBY Transcription Factor

R denotes replicates. There are two replicates (R1 and R2) for each developmental stage. S denotes Stage and there are 7 different developmental stages designated by S (1–7) which have been shown in Figure 1. Expression levels of transcripts are shown in RPKM (reads per kilobase of gene model per million mapped reads).

### ChIP-Seq libraries and detection of peaks for NAC and YABBY transcription factors

ChIP-Seq libraries were constructed using pooled cotyledons from soybean seedling developmental stage 4 and stage 5. After cross linking of cotyledon samples, chromatin complexes were isolated and sonicated for appropriate fragmentation for sequencing. We performed this experiment using different polyclonal antibodies raised against specific NAC and YABBY transcription factors using synthetic peptides (Table 2). The DNA-antibody complexes were precipitated and DNA was recovered from the complexes. Subsequently, ChIP-Seq libraries for NAC and YABBY transcription factors were constructed and high-throughput sequencing was performed. For NAC ChIP-Seq libraries, we obtained 21 million raw reads for the control library and 34 million raw reads for the antibody treated library (Table 3). Similarly sequencing of YABBY ChIP-Seq libraries generated 95 million raw reads for the control library and 86 million raw reads for the antibody treated library (Table 3).

**Table 2:** Synthetic peptides used to develop antibody against YABBY and NAC transcription factor

**TF name**	**Gene model**	**Amino acid position**	**Sequence of synthetic peptides**
			**Used for antibody production**
NAC	Glyma12g04380.1^*^	13-26	VRTGGKGTMRRKKK
	Glyma11g12170.1		
	Glyma11g12180.1^*^ Glyma01g18780.1		
YABBY	Glyma13g22620.1	99-112	TEERVVNRPPEKRQ
	Glyma17g12200.1		

All the antibodies are produced by GenScript Corporation. They used James and Wolf (JW) prediction algorithm to design synthetic peptides for the production of antibody against YABBY and NAC transcription factor.

^*^ Used for single amino acid difference between Glyma12g04380.1 and synthetic peptide at amino acid position 21 where Valine was replaced with Methionine (M).

**Table 3:** Summary of ChIP-Seq reads from four different libraries matched to the soybean genome

**TF Name**	**Condition**	**Raw reads**	**Genome matched reads**
NAC TF	Control	21548771	12017023
	Antibody treated	34605365	16780531
YABBY TF	Control	95047026	35218772
	Antibody treated	86685886	36005776

Sequencing of ChIP-Seq libraries produces raw read counts which were aligned to the soybean genome using ultrafast Bowtie aligner to get the number of genome matched reads. The experiment was conducted in two conditions, control library or antibody treated library.

**Figure 3:**
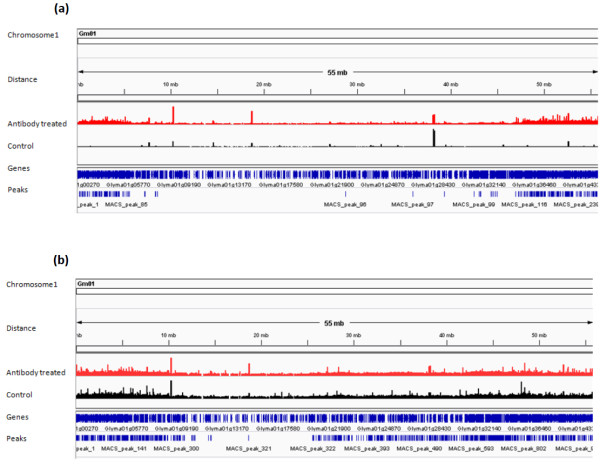
**Representative chromosomal view of soybean Chromosome 1 for NAC and YABBY transcription factor binding sites using Integrative Genomics Viewer (IGV) Genome browser.** Red peaks indicate potential transcription factor binding sites in antibody treated ChIP-Seq library. Black peaks are for the control ChIP-Seq library. Genes and location of peaks are shown in blue color. **(a)** Potential NAC transcription factor binding sites throughout the soybean chromosome 1. **(b)** Potential YABBY transcription factor binding sites along the soybean chromosome 1.

**Figure 4:**
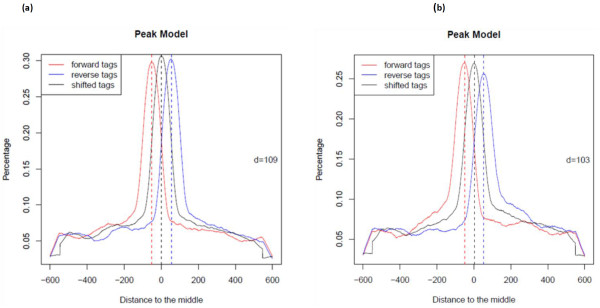
**MACS model for ChIP-Seq tags of NAC and YABBY transcription factor using ChIP-Seq data.** The red curve represents ChIP-Seq reads on the positive strand and the blue curve represents reads on the negative strand. The black curve illustrates the distribution of reads after shifting them towards 3′-end to get most likely protein-DNA interactions. **(a)** MACS software builds model for NAC transcription factor using the bimodal distribution of forward (+) and reverse (−) sequence tags. It calculates the estimated DNA fragment size, *d* =110 which is the distance between the peak in the forward and reverse strand. Then MACS shifts all the tags by *d*/*2* = 55 bp towards the 3′ ends. **(b)** Similarly MACS builds peak model for YABBY transcription factor. The estimated DNA fragment size, *d* =104 and MACS shifts all the tags by *d*/*2* = 52 bp towards the 3′ ends to get most likely TF-DNA interactions.

Millions of raw reads obtained from ChIP-Seq libraries were aligned to the reference soybean genome using the ultrafast Bowtie aligner [34] to obtain quantitative data for genome matched reads. There are numerous peak detection algorithms available for analyzing ChIP-Seq data sets. In this experiment MACS software [35] was used to call peaks representing enriched binding sites. The Bowtie alignment outputs for both control and antibody treated libraries were used together as input in the MACS software. For the NAC ChIP-Seq data set, MACS detected 8246 enriched peaks with p-value < 0.05 and for the YABBY ChIP-Seq data set, it detected 18064 (Table 4, Additional file 1 and Additional file 2). The distributions of MACS detected peaks in soybean chromosomes for both NAC and YABBY transcription factors were visualized using Integrative Genomics Viewer (IGV) software [36] (Figure 3). Additionally, MACS software builds the peak models for NAC and YABBY transcription factors separately using the bimodal distribution of forward (+) and reverse (−) sequence tags [35,37] (Figure 4). It calculates the estimated DNA fragment size, *d*, which is the distance between the peak in the forward and reverse strand. Then MACS shifts all the tags by *d*/*2* towards the 3′ ends to get the most likely protein-DNA interaction sites [10,11,35,37]. For the NAC ChIP-Seq dataset, MACS shifted all the tags 55 bp towards the 3′end to get most likely protein-DNA interactions whereas the shift was 52 bp for the YABBY transcription factor.

**Table 4:** Summary of output for ChIP-Seq data analysis using MACS

**TF name**	**Detected peaks**	**Peaks in intergenic region**	**Peaks associated with gene model**
NAC TF	8246	4743	3503
YABBY TF	18064	12430	5634

Using Bowtie alignments for both control and experimental conditions were analyzed using MACS software to detect peaks for the potential binding sites. The genomic locations of these peaks were identified using a custom made Python programing script.

### Locations of detected peaks and discovery of common motifs in the promoter regions

**Figure 5:**
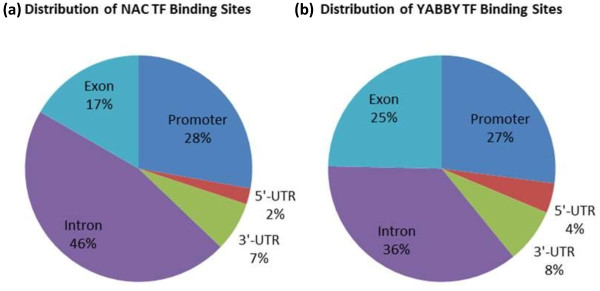
**Overview of NAC and YABBY transcription factor binding sites.** The genomic locations of peaks detected by ChIP-Seq were initially categorized into two classes, intergenic or gene model associated peaks. **(a)** The pie chart shows the distribution of ChIP-Seq peaks for NAC transcription factor binding sites associated with the gene models. **(b)** The pie chart shows the distribution of ChIP-Seq peaks for YABBY transcription factor binding sites associated with the gene models.

**Figure 6:**
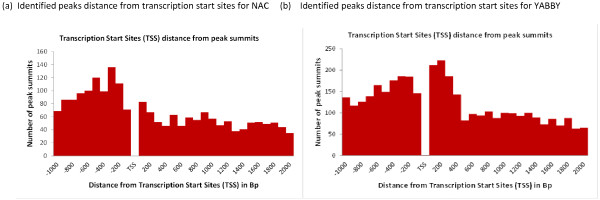
**NAC and YABBY Binding sites are highly enriched in the promoter region.** The distance between transcription start sites and MACS peak summits for the gene model associated peaks were determined. In this figure, peaks found up to 1000 bp upstream of TSS and 2000 bp downstream of TSS were included. **(a)** NAC binding sites are highly enriched in the promoter region. **(b)** YABBY binding sites are highly enriched in the promoter region.

**Figure 7:**
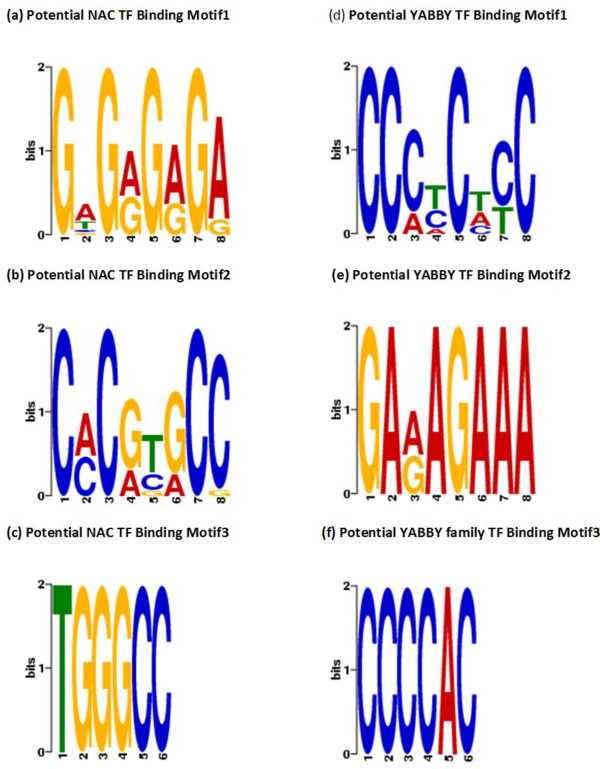
**Identified motifs for NAC and YABBY transcription factors using MEME.** The logos **(a-c)** show the three commonly motifs identified for NAC transcription factor using MEME software. Similarly, the logos **(d-f)** show the three commonly found motifs for YABBY transcription factors.

The genomic locations of MACS detected peaks were identified from the soybean gene annotation using a custom made Python programming script. We found that significant numbers of these peaks are located in the promoter region. For the YABBY ChIP-Seq dataset, 1526 peaks are located in the promoter region (Figure 5). Similarly for the NAC ChIP-Seq dataset, 974 peaks are located in the promoter region. Additionally we found that peaks are located in close proximity to the transcription start sites (TSS) (Figure 6). A motif search was performed using the most commonly used Multiple EM for Motif Elicitation (MEME) software [38]. For MEME analysis, we included those Glyma models whose promoter region contained at least one detected peak with a fold enrichment of 3 or more. The motif analysis discovered three separate motifs for the NAC and YABBY transcription factors (Table 5). For the NAC transcription factor, three commonly found motifs were G[AT]G[AG]G[AG]GA, C[AC]C[GA][TC][GA]CC and TGGGCC (Figure 7). The first one matched to a known zinc finger motif and the last two were identified as leucine zippers in the database of plant transcription factor binding motifs, JASPAR CORE plants [39,40]. Similarly the three most commonly found motifs for YABBY transcription factors are CC [CA][TC]C[TA][CT]C, GA[AG]AGAAA and CCCCAC (Figure 7). The first two motifs matched to a known zinc finger motif and the last one was an AP2 MBD-like motif.

**Table 5:** Discovery of motifs by MEME and their matches to known JASPAR CORE plant transcription factor binding site database

**TF Name**	**Motifs**	**Sequence**	**Matched to known motif**
NAC	Motif1	G[AT]G[AG]G[AG]GA	Zinc Finger
	Motif2	C[AC]C[GA][TC][GA]CC	Leucine Zipper
	Motif3	TGGGCC	Leucine Zipper
YABBY	Motif1	CC[CA][TC]C[TA][CT]C	Zinc Finger
	Motif2	GA[AG]AGAAA	Zinc Finger
	Motif3	CCCCAC	AP2 MBD-like

Motifs: Short DNA sequences for TFs binding. These motifs were discovered by MEME software.

ChIP-Seq tags which fall in the promoter region have been identified. For promoter associated peaks, 250 bp sequences from both sides of peak summits have been retrieved. These 500 bp sequences for associated Glyma models were given as input in MEME software to identify common motifs.

### ChIP-Seq coupled with RNA-Seq reveals candidate genes regulated by NAC and YABBY transcription factors

Our ChIP-Seq data identified 72 potential genes which are regulated by specific members of NAC transcription factor (Table 6 and Additional file 3). In similar way, we identified 96 potential candidate genes which are regulated by YABBY transcription factors (Table 7 and Additional file 4). We investigated the differential expression of NAC and YABBY regulated genes between stage 3 (before the functional transition) and stage 6 (after the functional transition) using our RNA-Seq data. RNA-Seq analysis revealed the expression in RPKM for these NAC and YABBY regulated candidate genes during different developmental stages. For differential expression analysis the DESeq package [41] efficiently identified differentially expressed genes between stage 3 and stage 6 with statistically significant P-values < 0.05. For the NAC transcription factor, we found that 10 candidate genes are up-regulated, 21 candidate genes are down-regulated and 41 candidate genes show no significant difference in their expression. Similarly for the YABBY transcription factor, we found that 19 candidate genes are up-regulated, 27 candidate genes are down-regulated and 50 candidate genes show no significant difference in their expression. The majority of these differentially expressed candidate genes are found to be involved in plant developmental processes.

**Table 6:** ChIP-Seq and RNA-Seq data reveals genes potentially regulated by NAC transcription factor

		**Stage 3**	**Stage 6**			
**Gene model**	**Motif**	**(RPKM)**	**(RPKM)**	**Regulation**	**P-value**	**Annotation**
Glyma01g00980.1	Motif1	7.7	2.1	Down	0.0013	RNA polymerase Rpb2
Glyma01g38630.1	Motif1	2.2	0.15	Down	1.23E-18	Cytochrome P450
Glyma01g42840.1	Motif3	8.5	10.7	Up	0.00032	Glutathione peroxidase
Glyma02g06730.1	Motif3	12.9	5.5	Down	0.01164	Homeobox domain containing protein
Glyma02g37130.1	Motif2,3	0.17	0.45	Up	0.00182	VQ motif containing protein
Glyma02g42990.1	Motif2	25.9	1.4	Down	9.27E-15	Polyketide cyclase / dehydrase
Glyma02g45000.1	Motif3	11.8	4.4	Down	0.01031	SNF2 family protein
Glyma03g05190.1	Motif2	2.8	0.23	Down	3.37E-07	Ferroportin1 (FPN1)
Glyma04g08540.1	Motif2	14.9	1.7	Down	9.49E-11	RBD/RNP domain containing protein
Glyma04g13490.1	Motif1	31.8	74.6	Up	1.79E-06	Pectin methylesterase inhibitor
Glyma06g02640.1	Motif1	5.4	1.1	Down	1.79E-08	Protein of unknown function
Glyma06g03070.1	Motif1	20.4	9.02	Down	0.03693	Transcription initiation factor IIF
Glyma06g16700.1	Motif1	19.9	30.7	Up	0.00936	GTP-binding protein
Glyma07g04690.1	Motif1	12.8	4.6	Down	0.00608	No Functional Annotation
Glyma07g05700.2	Motif1	6.2	27.5	Up	6.42E-12	No Functional Annotation
Glyma07g06660.1	Motif1	75.8	352	Up	2.01E-17	No Functional Annotation
Glyma07g07950.1	Motif3	16.3	6.8	Down	0.04714	DEAD/DEAH box helicase
Glyma07g38880.1	Motif2	10.8	2.1	Down	6.23E-05	Adenosine/AMP deaminase
Glyma08g23850.1	Motif3	20.4	3.8	Down	0.0001	RBD/RNP domain containing protein
Glyma10g32120.1	Motif1	1.1	0.21	Down	2.42E-07	No Functional Annotation
Glyma12g09540.1	Motif1	9.2	3.2	Down	0.01295	Sec23/Sec24 zinc finger
Glyma13g27730.1	Motif3	3.8	36.8	Up	6.69E-18	No Functional Annotation
Glyma13g42330.1	Motif3	315	1129	Up	8.19E-13	Lipoxygenase
Glyma15g18640.1	Motif1	10.7	6.4	Down	4.28E-06	No Functional Annotation
Glyma16g05210.1	Motif1	3.4	1.2	Down	0.02271	SET domain containing protein
Glyma16g27900.3	Motif1	1.1	0.01	Down	3.73E-09	No Functional Annotation
Glyma19g02030.1	Motif2	1.7	3.5	Up	0.00195	Phosphoglucose isomerase
Glyma19g42490.1	Motif1	1.6	0.31	Up	1.55E-07	No Functional Annotation
Glyma20g24810.1	Motif1	9.4	0.06	Down	5.66E-41	Cytochrome P450
Glyma20g27710.1	Motif2	0.97	0.37	Down	0.00576	Protein tyrosine kinase
Glyma20g28640.1	Motif1	46.9	8.5	Down	9.98E-09	Cupin domain containing protein

Complete table can be found in Additional file 3.

Binding motif number refer to those listed in Table 5. P-values were calculated by the DESeq package using the hit number from RNA-Seq libraries.

**Table 7:** ChIP-Seq and RNA-Seq data reveals genes potentially regulated by YABBY transcription factor

		**Stage 3**	**Stage 6**			
**Gene model**	**Motif**	**(RPKM)**	**(RPKM)**	**Regulation**	**P-value**	**Annotation**
Glyma01g03500.1	Motif2	1.4	4.05	Up	0.000246	Kelch motif containing protein
Glyma01g05750.1	Motif2	21.2	9.3	Down	0.014209	GDA1 nucleoside phosphatase
Glyma01g34490.1	Motif2	1.6	0.08	Down	4.41E-06	HSF-type DNA-binding protein
Glyma02g03890.1	Motif1,3	4.1	0.22	Down	2.69E-15	Armadillo/beta-catenin-like repeat
Glyma02g08840.1	Motif1	22.8	3.08	Down	6.59E-13	AP2 domain containing protein
Glyma02g35550.1	Motif2	1.7	4.5	Up	3.00E-07	Protein tyrosine kinase
Glyma03g39930.1	Motif3	14.6	8.2	Down	0.042524	Signal peptidase
Glyma04g33270.1	Motif2	0.70	0.13	Down	0.030654	No apical meristem (NAM) protein
Glyma05g36070.1	Motif1	4.7	1.8	Down	0.000671	GCC2 and GCC3
Glyma06g13910.1	Motif2	1.07	2.1	Up	0.00103	Auxin responsive protein
Glyma07g18350.1	Motif1	10.1	2.07	Down	0.000109	Fatty acid desaturase
Glyma08g19270.1	Motif3	21.3	6.01	Down	0.000737	Protein tyrosine kinase
Glyma10g38770.1	Motif3	0.66	2.4	Up	1.69E-11	PAZ domain containing protein
Glyma11g04900.1	Motif2	4.6	1.8	Down	0.020018	Leucine Rich Repeat protein
Glyma11g04950.1	Motif2	2.8	0.44	Down	0.009353	ACT domain containing protein
Glyma11g05920.1	Motif1	9.2	2.7	Down	0.000165	HLH DNA-binding domain protein
Glyma12g32590.1	Motif1	0.38	4.5	Up	2.05E-14	No Functional Annotation
Glyma12g32690.1	Motif1	49.4	0.9	Down	1.50E-36	No Functional Annotation
Glyma13g00380.1	Motif3	10.1	1.4	Down	3.36E-06	WRKY DNA -binding domain
Glyma13g00840.1	Motif1	18.3	2.8	Down	6.31E-11	E1-E2 ATPase
Glyma13g28450.1	Motif1	5.9	1.5	Down	2.03E-08	Sugar transporter
Glyma13g31010.1	Motif2	15.1	0.50	Down	4.31E-25	AP2 domain containing protein
Glyma14g36810.1	Motif2	1.6	3.4	Up	7.07E-05	lectin domain containing protein
Glyma15g13830.1	Motif3	38.1	13.8	Down	3.10E-05	GTPase of unknown function
Glyma18g02280.3	Motif3	1.3	4.2	Up	0.000148	No Functional Annotation
Glyma18g42700.1	Motif2	3.9	0.08	Down	2.26E-15	Protein tyrosine kinase
Glyma19g32880.1	Motif1	11.8	0.72	Down	7.91E-23	Cytochrome P450
Glyma20g03420.1	Motif2	8.06	2.3	Down	7.47E-06	GC-rich DNA-binding factor protein
Glyma20g04490.1	Motif2	17.6	16.5	Down	0.016291	lipoprotein A (RlpA)-like protein
Glyma20g24990.2	Motif2	54.5	128.6	Up	2.88E-06	No Functional Annotation

Complete table can be found in Additional file 4.

Binding motif number refer to those listed in Table 5. P-values were calculated by the DESeq package using the hit number from RNA-Seq libraries.

## Discussion

The regulation of gene expression by transcription factors is a quite complex and coordinated process. Recently the ENCODE (ENCyclopedia of DNA Elements) project has generated chromatin immunoprecipitation followed by high-throughput sequencing (ChIP-seq) data sets for a large number of transcription factors using different human cell lines to identify genome-wide functional and regulatory DNA elements [42,43]. Although better understood in model plants such as *Arabidopsis* and rice [5,44,45] knowledge is scarce in most other plants. A number of transcription factor binding sites have been identified in *Arabidopsis* using recently developed techniques such as Chromatin Immunoprecipitation Sequencing (ChIP-Seq) [46-49]. Soybean is a polyploid crop having a complex and large genome [15]. To date, there are no reports of identification of soybean transcription factor binding sites using the high throughput ChIP-Seq technique. In order to identify two developmentally important transcription factors binding sites during soybean seedling development, we used a combination of experimental and bioinformatics approaches. In this study, ChIP-Seq and RNA-Seq were used to dissect the gene regulatory networks for NAC and YABBY transcription factors during soybean seedling development. We constructed seven RNA-Seq libraries using cotyledons from seven different seedling developmental stages separately to see the expression level of transcription factors and their co-regulated genes. Later we constructed separate ChIP-Seq libraries for specific NAC and YABBY transcription factors using pooled cotyledons from soybean seedling developmental stage 4 and stage 5 when the cotyledons undergo a functional transition from non-photosynthetic storage tissues to metabolically active photosynthetic tissues.

The NAC transcription factor is a plant specific transcription factor family which plays important roles in plant growth, development and stress responses [23-25]. *Glycine max* has more than 100 different NAC proteins [16,23]. Although NAC transcription factor family is quite large, our RNA-Seq data showed that there are only four specific members of NAC family expressed and showed a clear expression pattern during soybean seedlings development (Table 1 and Figure 2). Additionally, we performed the multiple sequence alignment of these four members of NAC family and found a high homology among their sequences (Additional file 5). These four members of NAC family possess that short peptide sequence used for developing the antibody and they are closely related. For the ChIP-Seq experiment, we used germinating cotyledons from stage 4 and stage 5 which are the transition stages. Thus, our antibody is specific for these four members of the NAC family since they show high homology in their sequences and are the only members expressed during the physiological transition at stage 4 and stage 5. The analysis of ChIP-Seq libraries (control and antibody treated) for the NAC transcription factor using MACS software detected 8246 highly enriched peaks with statistical significance P < 0.05. A significant number of these peaks are associated with soybean gene models. We found that 974 peaks are located in the promoter region of soybean gene models. For MEME analysis, we selected those Glyma models whose promoter region contains at least one detected peak with a fold enrichment of 3 or more over the control. We found three common DNA binding motifs, two of them matched to leucine zipper and one matched to a zinc finger (Figure 7 and Table 5). Previously it had been reported in *Arabidopsis* that the NAC transcription factor binding site contains the consensus DNA sequence (CGT[GA]) [26]. One of our identified common motifs was C[AC]**C**[**G**A][**T**C][**GA**]CC which contains the previously identified motif in *Arabidopsis*, thus corroborating our discovery of DNA binding motifs for the NAC transcription factor in soybean.

To dissect the gene regulatory network of a particular transcription factor, it is important to study the expression of co-regulated genes. In this study, we have identified 72 genes potentially regulated by a NAC transcription factor based on our ChIP-Seq and RNA-Seq data (Table 6 and Additional file 3). Using our developmental stage specific RNA-Seq data, we investigated their expression levels. Our particular interest was on developmental stage 3 which is before the functional transition and developmental stage 6 which is after the functional transition. DESeq analysis showed differential expression of a number of candidate genes at p-value < 0.05. We focused on 10 up-regulated and 21 down-regulated genes to see the level of expression difference in between stage 3 and stage 6. From the RNA expression data, the highest level of expression difference was found with genes annotated as lipoxygense, pectin methylesterase inhibitor (PMEI), DEAD/DEAH box helicase and Homeobox associated proteins. DESeq analysis also showed very low p-values corresponding to these gene models indicating they are significantly differentially expressed.

Among those highly differentially expressed genes, lipoxygenase has been proposed to be involved in reserve lipid mobilization during soybean seed germination [17,50,51]. Our RNA-Seq data showed that the lipoxygenase gene is up-regulated. Once germination is triggered, lipids need to be mobilized by the action of lipoxygenase and ultimately triacylglycerols are degraded to act as a carbon and energy source for the developing seedlings. Another candidate gene encodes a cupin domain containing protein, which has been reported to be involved in seed germination and early seedling development [52,53]. We found that the cupin gene is down-regulated by the specific NAC transcription factor. This might be due to the fact that after the functional transition the seedling tends to shift towards a normal photosynthetic cycle instead of the glyoxylate cycle. In this study, we found pectin methylesterase inhibitors (PMEIs) are regulated by the specific members of NAC transcription factor. Pectin, one of the main components of the plant cell wall, is continually modified and remodeled during plant growth and development [54,55]. Pectin methylesterases (PMEs) catalyse the demethylesterification of cell wall pectins [55,56]. In many developmental processes, PMEs are regulated by either differential expression or posttranslational control by pectin methylesterases inhibitors (PMEIs) [57]. These PMEI inhibitors play significant roles in plant growth, cell division, and expansion [56,57]. We identified PMEI as a NAC regulated potential candidate gene and the expression of PMEI gene is up-regulated by this NAC transcription factor, indicating that PMEI reduces the activity of PMEs during later stages of seedling development.

**Figure 8:**
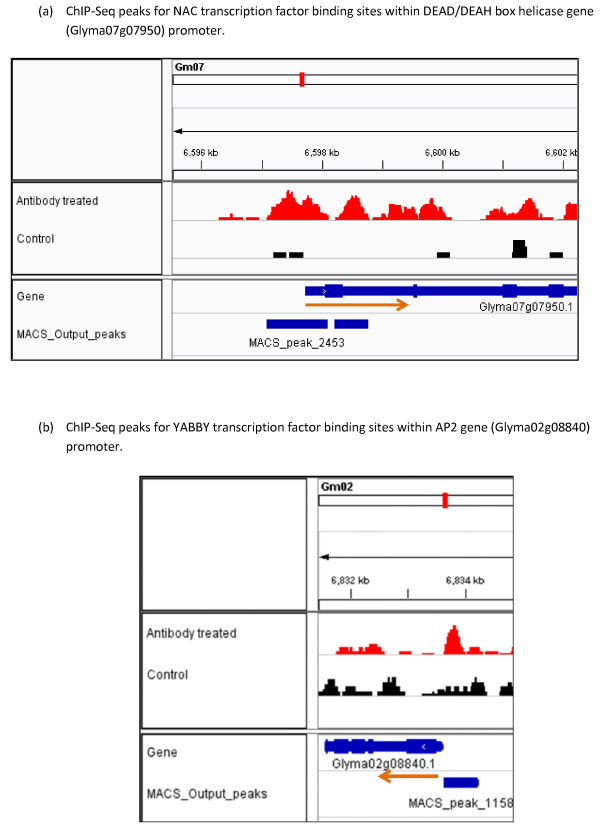
**Visualization of ChIP-Seq results for two representative NAC and YABBY regulated genes by Integrative Genomics Viewer (IGV).** Red peaks indicate potential transcription factor binding sites in antibody treated ChIP-Seq library. Black peaks are for the control ChIP-Seq library. Genes and location of peaks are shown in blue color. The red arrow indicates the direction of transcription. **(a)** ChIP-Seq peaks for NAC transcription factor binding sites within DEAD/DEAH box helicase gene (Glyma07g07950) promoter. **(b)** ChIP-Seq peaks for YABBY transcription factor binding sites within AP2 gene (Glyma02g08840) promoter.

Another important gene regulated by the NAC transcription factor is the DEAD/DEAH box helicases which are ubiquitous enzymes that catalyze the unwinding of energetically stable duplex DNA (DNA helicases) or duplex RNA secondary structures (RNA helicases) [58-60]. Most helicases are members of the DEAD-box protein superfamily and play essential roles in basic cellular processes such as DNA replication, repair, recombination, transcription, ribosome biogenesis and translation initiation [59,61]. Therefore, helicases might be playing an important role in regulating plant growth and development. Our ChIP-Seq results showed the potential NAC transcription factor binding sites in the promoter region of DEAD/DEAH box helicase gene (Figure 8a). ChIP-Seq and RNA-Seq analysis together showed that the DEAD/DEAH box helicase gene is down-regulated by the NAC transcription factor. Using our RNA-Seq data, we found that a particular Homeotic (HOX) gene was down- regulated by the NAC transcription factor. The plausible explanation for this down regulation might be that HOX genes are known to be involved in flower development [62,63] and thus it is expected to be down-regulated during seedling development. Taken together, these results indicate that NAC transcription factors act in multiple pathways to regulate gene expression that facilitate the functional transition of the cotyledons during legume seedling growth.

The YABBY transcription factor family is a group of plant specific transcription factors which play important roles in organ polarity determination [28-30]. Members of the YABBY gene family are characterized by two conserved domains, a C2C2 zinc finger-like domain towards the amino terminus and a helix-loop-helix, which is called the YABBY domain, with sequence similarity to the first two helices of the HMG box towards the carboxyl end of the protein [19,20,32]. *Glycine max* has multiple isoforms of the YABBY transcription factor; however there is not much known about the molecular mechanisms for this transcription factor specifically its DNA binding sites and co-regulated genes. Our RNA-Seq data revealed that there are ten members of the YABBY transcription factor family expressed during the physiological transition. We performed a multiple sequence alignment among the ten members of the YABBY transcription factors. Two of them showed high sequence homology and they are closely related (Additional file 6). Only these two members possess the short peptide sequence used for developing the antibody. Thus, our antibody is specific for those two members of the YABBY family that showed the clear expression pattern during the functional transition. The analysis of ChIP-Seq libraries (control and antibody treated) for the YABBY transcription factor detected 18064 highly enriched peaks with statistical p-value < 0.05. A significant number of these peaks are associated with gene models. We found that 1526 peaks are located in the promoter region of soybean gene models. We found three common DNA binding motifs by MEME analysis; two of them match to the zinc finger motif and one matches to the AP2 MBD-like motif (Figure 7 and Table 5).

In this study, we have identified YABBY regulated genes based on our ChIP-Seq and RNA-Seq data. We found that there are 96 genes potentially regulated by the YABBY transcription factor (Table 7 and Additional file 4). Using our developmental stage specific RNA-Seq data, we have investigated their expression levels before the functional transition (developmental stage 3) and after the functional transition (developmental stage 6). DESeq analysis showed differential expression of a number of candidate genes at p-value < 0.05. We carefully looked at the expression data for the 19 up-regulated and 27 down-regulated genes to see the level of expression difference between stage 3 and stage 6. From the RNA expression data, the highest level of expression difference was found with genes annotated as protein AP2 (APETALA2) transcription factor, fatty acid desaturase and WRKY DNA binding domain protein as well as some other genes with no functional annotation. Among them AP2 is a very well-known transcription factor unique to plants, whose distinguishing characteristic is its AP2 DNA-binding domain [64-66]. It plays a key role in several developmental processes like floral organ identity determination and control of leaf epidermal cell identity and is under strict regulation during developmental processes [65,67-70]. Our ChIP-Seq results showed the potential YABBY transcription factor binding sites in the promoter region of AP2 gene (Figure 8b). Since it is mainly involved in flower developmental, it is down-regulated at the later stages of seedling development.

The existence of repeated DNA-binding domains not unique to AP2 transcription factors, the WRKY family of transcription factors also may contain a repeated DNA-binding domain [71]. Our RNA-Seq data revealed that there are 53 genes encoding WRKY transcription factors expressed at level (≥ 10 RPKM) in at least one of seven stages of soybean seedling development (Additional file 7). Based on our ChIP-Seq result, a specific WRKY Transcription factor is one among five major candidates regulated by YABBY transcription factor. Previous reports show that the WRKY transcription factor has been involved in the numerous plant developmental processes [72-76]. Specific members of WRKY transcription factor family are playing important role in seed development [72,73]. However it is quite difficult to pinpoint the regulation by the WRKY transcription factor since there are large numbers of WRKY transcription factors in soybean. Another YABBY regulated candidate gene is Fatty Acid Desaturase (FAD). It encodes the main enzyme responsible for polyunsaturated lipid synthesis in developing seeds of oil crops [77,78]. Our results showed that fatty acid desaturase was down-regulated by the YABBY transcription factor in agreement with lipid catabolism being more preferable during seedling development than lipid biosynthesis which needs fatty acid desaturase. Our ChIP-Seq results reveal the genome-wide view of binding sites for the YABBY transcription factor and RNA-Seq demonstrates the resultant changes in expression of regulated genes that influence the physiological transition of the soybean cotyledon from a storage tissue to a metabolically active tissue during seedling growth.

## Conclusion

ChIP-Seq demonstrates promising potential as a new tool in understanding genome-wide binding sites for transcription factors and transcriptional gene regulatory networks. Our genome-wide identification of NAC and YABBY transcription factor binding sites using antibodies to synthetic peptides representing these rare abundance transcription factors will help to better understand the transcriptional gene regulatory network during the functional transition of cotyledons from a storage tissue to a metabolically active photosynthetic tissue. The discovery of common DNA binding motifs and identification of regulated genes opens a new avenue to pinpoint the molecular mechanisms of these two important transcription factors during seedling growth. Combining ChIP-Seq and RNA-Seq results advances understanding of the underlying genetic mechanisms involved in the functional transition as well as their regulation and control systems throughout the soybean seedling developmental process.

## Methods

### Plant materials and growth conditions

Four soybean (Glycine max cv. Williams) seeds were planted per small pot (4.5 inches) containing Universal SB300 soil mix. A total of 25 pots (100 seeds) were initially used to collect and pool 6 individual cotyledon samples per developmental stage. Plants were grown for approximately 7–8 days with regular watering. A biological replicate was performed with another 25 pots to collect tissues in a similar way. Seven different stages during the development of soybean seedlings were defined based on time, size of radicles, hypocotyls, roots and appearance of germinating cotyledons. Stage 1: Imbibed seed for 24 hours; pre-emerging hypocotyls. Stage 2: Yellow cotyledons; emerging radicle 8–10 mm long. Stage 3: Yellow cotyledons with slightly green edges; hypocotyls15-20 mm long. Stage 4: Yellow-green cotyledons; hypocotyls 30–35 mm long. Stage 5: Yellow-green cotyledons above the ground; primary roots starting to develop. Stage 6: Mostly green cotyledons above the ground; growing straight from the hypocotyl. Stage 7: Fully green cotyledons; plants 6–7 cm long above the ground; the root system fully developed; cotyledons upright; unifoliolate exposed. For the RNA-Seq experiment, cotyledons from each of these developmental stages were collected and then frozen in liquid nitrogen. Subsequently the tissue was freeze dried and stored at −80°C. For ChIP-Seq experiment, fresh cotyledons from stage 4 and stage 5 were used.

### RNA-Seq library construction and data analysis

Total RNA was extracted separately for seven different developmental stages from freeze dried cotyledons using a modified McCarty method [79] using phenol-chloroform extraction and lithium chloride precipitation. A biological replicate was performed to extract RNA in a similar way. Library construction and high-throughput sequencing were carried out by the Illumina HiSeq2000 at the Keck Center, University of Illinois at Urbana-Champaign. The 100 bp RNA-Seq reads were mapped to the 78,773 high and low confidence soybean gene models [33] using the ultrafast Bowtie aligner [34] with up to 3 mismatches. RNA-Seq data was normalized in reads per kilobase of gene model per million mapped reads (RPKM) [14]. The DESeq package [41] was used to determine differential expression between developmental stage 3 and stage 6 and calculate p-values. If the p-value was < 0.05, we considered that gene as significantly differentially expressed gene between two developmental stages. The expression could be up-regulated or down-regulated based on the corresponding RPKM values.

### ChIP-Seq library construction

Cotyledons from soybean seedling developmental stage 4 and stage 5 were collected for the ChIP-Seq experiment performed using previously described methods [80,81]. Briefly, 0.08 g of fresh weight of soybean cotyledons from stage 4 or stage 5 were cross sectioned with a razor blade and then cross linked with 1% formaldehyde under vacuum. Immediately the samples were ground to powder in liquid nitrogen. The chromatin complexes were isolated following previously established protocols [80,81]. Later, the chromatin was sonicated to shear DNA into 200–600 bp fragments using the 15% power setting and fifteen times for 20 second pulses using a Branson digital probe sonifier. Sample containing tubes were kept on ice while the sonication was performed. Subsequently, the sonicated DNA was incubated with a polyclonal antibody developed against the YABBY or NAC transcription factors. All the antibodies were produced by GenScript Corporation. They used the Jameson and Wolf (JW) prediction algorithm [82] to design synthetic peptides for the production of antibody against YABBY and NAC transcription factor [Table 2]. Separate controls which were not treated with antibody, but used preimmune sera, were used for each experiment. Then DNA-antibody complexes were precipitated following standard protocol [81,83] and DNA was recovered by dissociating the complexes. ChIP-Seq library construction and high-throughput sequencing was carried out by the Illumina HiSeq2000 at the Keck Center, University of Illinois at Urbana-Champaign.

### ChIP-Seq data analysis

Sequencing of ChIP-Seq libraries produced millions of raw reads which were aligned to the reference soybean genome using the ultrafast Bowtie aligner [34] to get the number of genome matched reads. The length of our sequence reads was 100 bp and we allowed 3 mismatches for Bowtie alignment. The experiment was conducted in two conditions, the control library and the antibody treated library. MACS software [35] with specific parameters (bandwidth 300 bp; mfold, 30; P-value of 1.00e-05) was used to call peaks representing enriched binding sites. The Bowtie alignment output for both control and antibody treated libraries was used together as input to the MACS software to detect a number of peaks for the potential binding sites for the YABBY or NAC transcription factors separately. Since ChIP-DNA fragments are equally likely to be sequenced from both ends, the tag density around a true binding site should show a bimodal enrichment pattern, with forward strand tags enriched upstream of binding sites and reverse strand tags enriched downstream of binding sites [10,11,35,37]. MACS software takes advantage of this bimodal pattern to empirically model the shifting size to better locate the precise binding sites. It randomly samples 1,000 of these high-quality peaks, separates their forward (+) and reverse (−) tags, and aligns them by the midpoint between their forward and reverse tag centers [35,37]. MACS calculated estimated DNA fragment size, d which is the distance between the peak in the forward and reverse strand. Then MACS shifts all the tags by *d*/*2* toward the 3′ ends to get the most likely protein-DNA interaction sites [10,11,35,37]. Then the genomic locations of these peaks were identified from the soybean gene annotation file from the Phytozome database [33] using a custom made Python programming script. Using that programming script, all binding peaks were sorted based on the following criteria: (1) if a binding site resides in the gene body, it will be further categorized according to its location in the gene body (i.e., 5′-untranslated region, exon, intron, or 3′-untranslated region); (2) if a binding site is localized in the 1000-bp region upstream of the transcription start site of a gene, it is classified as a binding site in the promoter region in our study; the binding sites not selected by the above criteria were defined as the binding sites in the intergenic regions. The outputs of the analysis, specifically the detected peaks were visualized in the Integrative Genomics Viewer genome browser [36].

### Motif search

A motif search was performed using the most widely used MEME software [38]. For MEME analysis, gene models were selected based on the location of detected peaks and fold enrichment. In this analysis, we included those gene models whose promoter region contains at least one detected peak and a fold enrichment of 3 or more. For promoter associated peaks, 250 bp sequences from both sides of peak summits were retrieved. These 500 bp sequences for associated gene models were given as input in MEME software to identify common motifs. Some of these identified motifs were matched to known motifs in the plant transcription factor binding sites database, JASPAR CORE plants [39,40].

### Data availability

The high-throughput sequencing data for ChIP-Seq libraries are available under NCBI-GEO [84] series accession no. GSE42422. In addition, RNA-Seq data for seven developmental stages with two biological replicates are available under NCBI-GEO [83] series accession no. GSE42550.

## Competing interests

The authors declare that they have no competing interests.

## Authors’ contributions

MS designed experiments, performed RNA extractions, conducted ChIP-Seq experiment, analyzed RNA-Seq and ChIP-Seq data and drafted the manuscript. LOV designed initial approach, obtained funding, led and coordinated the project, and edited the manuscript. All authors read and approved the final manuscript.

## Supplementary Material

Additional file 1 Peaks detected for NAC transcription factor binding sites by MACS software. Click here for file

Additional file 2 Peaks detected for YABBY transcription factor binding sites by MACS software. Click here for file

Additional file 3 Complete list of candidate genes potentially regulated by NAC transcription factor during soybean seedling development. Click here for file

Additional file 4 Complete list of candidate genes potentially regulated by YABBY transcription factor during soybean seedling development. Click here for file

Additional file 5 Multiple sequence alignment of four members of NAC family expressed during the functional transition of the cotyledons. Click here for file

Additional file 6 Multiple sequence alignment of ten members of YABBY family expressed during the functional transition of the cotyledons. Click here for file

Additional file 7 List of 53 gene models encoding WRKY transcription factors expressed at level ( ≥ 10 RPKM) in at least one of seven stages of soybean seedling development. Click here for file
