# Supplementary material for: Genome-wide identification of binding sites for NAC and YABBY transcription factors and co-regulated genes during soybean seedling development by ChIP-Seq and RNA-Seq
Source: BMC Genomics. 2013 Jul 16;14:477. doi: 10.1186/1471-2164-14-477 (PMC3720225; doi:10.1186/1471-2164-14-477)
Supplement: Additional file 5 — Multiple sequence alignment of four members of NAC family expressed during the functional transition of the cotyledons. [file 1471-2164-14-477-S5.pdf]

## Additional file 5: Multiple sequence alignment of four members of NAC family expressed during the functional transition of the cotyledons

```
Glyma12g04383.1      MNREKLMKMAGSVRTGGKGTVRRKKKAVHKTTTTDDKRLQSTLKRIGVNAIPAIEEVNIF 60
Glyma11g12180.1      MNREKLMKMAGSVRTGGKGTVRRKKKAVHKTTTTDDKRLQSTLKRIGVNAIPAIEEVNIF 60
Glyma11g12170.1      MNREKLMKMAGSVRTGGKGTMRRKKKAVHKTTTTDDKRLQSTLKRIGVNAIPAIEEVNIF 60
Glyma01g18780.1      MDVEKLKKMASSVRTGGKGTMRRKKKAVHKTTTTDDKRLQSTLKRIGVNAIPAIEEVNIF 60
                     *: *** ***.**********:**************************************

Glyma12g04383.1      KDDVVIQFLNPKVQASIAANTWVVSGSPQTKKLQDILPSIIHQLGPDNLENLKKLAEQFQ 120
Glyma11g12180.1      KDDVVIQFLNPKVQASIAANTWVVSGSPQTKKLQDILPSIIHQLGPDNLENLKKLAEQFQ 120
Glyma11g12170.1      KDDVVIQFLNPKVQASIAANTWVVSGSPQTKKLQDILPSIIHQLGPDNLENLKKLAEQFQ 120
Glyma01g18780.1      KDEIVIQFLNPKVQASIAANTWVVSGSPQTKKLQDILPNIIHQLGPDNLENLKKLAEQFQ 120
                     **::**********************************.*********************

Glyma12g04383.1      KQAPEGAAGSTTAQEENDDD-VPELVPGQDFETAAEETKAAS 161
Glyma11g12180.1      KQAPEGATGSTTAQEENDDDDVPELVPGQDFETAAEETKAAS 162
Glyma11g12170.1      KQAPEGAAGSTTAQEENDDDDVPELVPGQDFETAAEETKAAS 162
Glyma01g18780.1      KQVPEAGDGATAAQE-DDDDAVPDLVPGETFETAAEETKS-- 159
                     **.**.. *:*:*** :*** **:****: *********:
```

Our RNA-Seq data revealed that four specific members of NAC transcription factor family expressed during functional transition (stage-4 and stage-5) of soybean seedling development. We used pooled cotyledons of stage 4 and stage 5 for ChIP-Seq experiment. These four members of NAC family possess that synthetic peptide sequence (black rectangle) used for developing the antibody and they are closely related. Thus, our antibody is specific for these four members of NAC family since they show high homology in their sequence and are the only members expressed during physiological transition at stage 4 and stage 5.
