# Supplementary material for: Genome-wide identification of binding sites for NAC and YABBY transcription factors and co-regulated genes during soybean seedling development by ChIP-Seq and RNA-Seq
Source: BMC Genomics. 2013 Jul 16;14:477. doi: 10.1186/1471-2164-14-477 (PMC3720225; doi:10.1186/1471-2164-14-477)
Supplement: Additional file 6 — Multiple sequence alignment of ten members of YABBY family expressed during the functional transition of the cotyledons. [file 1471-2164-14-477-S6.pdf]

Additional file 6: Multiple sequence alignment of ten members of YABBY family expressed during the functional transition of the cotyledons

```
Glyma12g31670.1      ---MSMDMMATER--------VCYVHCNFCNTTLAVSVPCSSLLTIVTVRCGHCANLLTV 49
Glyma13g38750.1      ---MSMDMMATER--------VCYVHCNFCNTTLAVSVPCSSLLTIVTVRCGHCANLLTV 49
Glyma12g10210.1      ---MSMEMMATER--------VCYVHCNFCNTILAVSVPYSSLLTIVTVRCGHCANLLSV 49
Glyma06g46560.1      MYVLLCQCVYISE--------SVLVLAFYFLVYILVSVPYSSLLTIVTVRCGHCANLLSV 52
Glyma17g12200.1      -MSSCSIDVAPEQ--------LCYIPCNFCNIVLAVSVPCSSLFDIVTVRCGHCTNLWSV 51
Glyma13g22620.1      -MSSCSIDVAPEQ--------LCYIPCNFCNIVLAVSVPCSSLFDIVTVRCGHCTNLWSV 51
Glyma03g03500.1      -MSSSSTSFSPDQQHLSPSDQLCYVHCNFCDTVLAVSVPCTSLFKNVTVRCGHCTNLLSV 59
Glyma01g33370.1      -MSSSSTSFSPDQ-HLSPSDQLCYVHCNFCDTVLAVSVPCTSLFKTVTVRCGHCTNLLSV 58
Glyma05g04260.1      -MSSSSTTLSLDH--LPPSEQLCYVHCNICDTVLAVSVPCTSLFKTVTVRCGHCTNLLPV 57
Glyma17g14710.1      -MSSSSSTLSLDH--LPPSEQLCYVHCNICDTVLAVSVPCTSLFKTVTVRCGHCTNLLPV 57
                               .  ..           : .      : **** :**:   *********:**  .*

Glyma12g31670.1      NMGASLQTFPSQDTT---------QLQRQHLSVQEACS-KELGSSS----KCKSF-ETVD 94
Glyma13g38750.1      NMGASLQTFPSQDTT---------QLQRQHLSVQEACS-KELGSSS----KCKTF-ETVD 94
Glyma12g10210.1      NMGASLQAFPPQDP----------QSQKQHLSFQEPSS-KELGSSS-KCSKIAPF-EAVE 96
Glyma06g46560.1      NMGASLQAFPPQDP----------QSQKQLLSFEEPSSCKELGSSSSKCNKIAPFHEAVE 102
Glyma17g12200.1      NMAAAFQSLSWQDV--------------QGPGQCNPEYRIDTGSTS-KCNNRIAMRAPTT 96
Glyma13g22620.1      NMAAAFQSLSWQDV--------------QGSGHCNPEYRIDTGSTS-KCNNRIAMRAPTT 96
Glyma03g03500.1      NMRGLLLPSANQLHLGHTFFTP-QNLMEEIRNAPSTNIMMNQ--LPNPNDLVMSTMRGGP 116
Glyma01g33370.1      NMRGLLLPSANQLHLGHSFFTP-QNLLEEIRNAPSTNMMMNQ--LPNPNDLVMSTMRGGP 115
Glyma05g04260.1      NMRGLLMPSPTQFHLGHSFFSPSHNLLEEIPN-PSPNFLMNQTNLSASNEFSMP-ARIAA 115
Glyma17g14710.1      NMRGLLMPSPTQFHLGHSFFSPSHNLLEEIPN-PTPNFLMNQTNFSASHEFSMP-ARTAA 115
                     ** . : . . *                 :  .    .     :    .       .

Glyma12g31670.1      HEQQPRIPPIRPPEKRQRVPSAYNRFIKEEIQRIKASNPDISHREAFSTAAKNWAHFPHI 154
Glyma13g38750.1      HDQQPRIPPIRPPEKRQRVPSAYNRFIKEEIQRIKASNPDISHREAFSTAAKNWAHFPHI 154
Glyma12g10210.1      HEL-PRIPPIRPTEKRHRVPSAYNRFIKEEIQRIKASNPDISHREAFSSAAKNWAHFPHI 155
Glyma06g46560.1      HEQ-PRIPPIRPTEKRHRVPSAYNRFIKEEIQRIKASNPDISHREAFSSAAKNWAHFPHI 161
Glyma17g12200.1      HVTEERVVN-RPPEKRQRVPSAYNQFIKEEIQRIKANNPDISHREAFSTAAKNWAHFPHI 155
Glyma13g22620.1      HVTEERVVN-RPPEKRQRVPSAYNQFIKEEIQRIKANNPDISHREAFSTAAKNWAHFPHI 155
Glyma03g03500.1      EETPKPPSANRPPEKRQRVPSAYNRFIKDEIQRIKAGNPDISHREAFSAAAKNWAHFPHI 176
Glyma01g33370.1      EETPKPPSANRPPEKRQRVPSAYNRFIKDEIQRIKAGNPDISHREAFSAAAKNWAHFPHI 175
Glyma05g04260.1      DELPR-PIMNRPPEKRQRVPSAYNRFIKDEIQRIKSVNPDITHREAFSAAAKNWAHFPHI 174
Glyma17g14710.1      DELPRPPITNRPPEKRQRVPSAYNRFIKDEIQRIKSVNPDITHREAFSAAAKNWAHFPHI 175
                     .         **.***:*******:***:******: ****:******:***********

Glyma12g31670.1      HFGLK---LDGNKQAKLD-QGDGTQKSNGFY---------- 181
Glyma13g38750.1      HFGLK---LDGNKQAKLD-QGDGTQKSNGFY---------- 181
Glyma12g10210.1      HFGLKNLKLDGNKQEKLD-QGEGAEKSNGFY---------- 185
Glyma06g46560.1      HFGLKNLKLDGNKQEKLD-QGEGAEKSNGFY---------- 191
Glyma17g12200.1      HFGLM---LESNNQAKMDNVSEKHLMPRAALLNK------- 186
Glyma13g22620.1      HFGLM---LESNNQVKMENVSEKHLMSRAALLNK------- 186
Glyma03g03500.1      HFGLMPDNQPVKKANVR-QEAEDVLMKDGFFAPANVGVSPY 216
Glyma01g33370.1      HFGLMPDNQPVKKANVR-QEAEDVLMKDGFFAPANVGVSPY 215
Glyma05g04260.1      HFGLMPD-QTVKKTNVCQQEGEEVLMKDGFYASANVGVSPY 214
Glyma17g14710.1      HFGLMPD-QTVKKTNVCQQDGEEVLMKDGFYASANVGVSPY 215
                     ****       ::       .:      .
```

Our RNA-Seq data revealed that ten specific members of YABBY transcription factor family expressed during functional transition (stage-4 and stage-5) of soybean seedling development. Two of them showed high sequence homology and they are closely related. These two members of YABBY family possess the short peptide sequence (black rectangle) used for developing the antibody. Thus, our antibody is specific for those two members of YABBY family which showed a clear expression pattern during the functional transition of the cotyledons.
